# Chronic Hepatitis C-Related Cirrhosis Hospitalization Cost Analysis in Bulgaria

**DOI:** 10.3389/fmed.2017.00125

**Published:** 2017-08-07

**Authors:** Maria Dimitrova, Kaloyan Pavlov, Konstantin Mitov, Jordan Genov, Guenka Ivanova Petrova

**Affiliations:** ^1^Faculty of Pharmacy, Medical University, Sofia, Bulgaria; ^2^University Hospital “Queen Joanna-ISUL”, Sofia, Bulgaria

**Keywords:** chronic HCV infection, HCV-related cirrhosis, hospitalizations, cost analysis, burden

## Abstract

**Objective:**

HCV infection is a leading cause of chronic liver disease with long-term complications—extensive fibrosis, cirrhosis, and hepatocellular carcinoma. The objective of this study is to perform cost analysis of therapy of patients with chronic HCV-related cirrhosis hospitalized in the University Hospital “Queen Joanna-ISUL” for 3-year period (2012–2014).

**Methods:**

It is a prospective, real life observational study of 297 patients with chronic HCV infection and cirrhosis monitored in the University Hospital “Queen Joanna-ISUL” for 3-year period. Data on demographic, clinical characteristics, and health-care resources utilization (hospitalizations, highly specialized interventions, and pharmacotherapy) were collected. Micro-costing approach was applied to evaluate the total direct medical costs. The points of view are that of the National Health Insurance Fund (NHIF), hospital and the patients. Collected cost data are from the NHIF and hospitals tariffs, patients, and from the positive dug list for medicines prices. Descriptive statistics, chi-squared test, Kruskal–Wallis, and Friedman tests were used for statistical processing.

**Results:**

76% of patients were male. 93% were diagnosed in grade Child-Pugh A and B. 97% reported complications, and almost all developed esophageal varices. During the 3 years observational period, patients did not change the critical clinical values for Child-Pugh status and therefore the group was considered as homogenous. 847 hospitalizations were recorded for 3 years period with average length of stay 17 days. The mortality rate of 6.90% was extremely high. The total direct medical costs for the observed cohort of patients for 3-year period accounted for 1,290,533 BGN (€659,839) with an average cost per patient 4,577 BGN (€2,340). Statistically significant correlation was observed between the total cost per patient from the different payers’ perspective and the Child-Pugh cirrhosis score.

**Conclusion:**

HCV-related cirrhosis is resource demanding and sets high direct medical costs as it is related with increased hospitalizations and complications acquiring additional treatment.

## Introduction

Chronic hepatitis C infection is a major health and economic concern as it is a leading cause of chronic liver disease with serious long-term complications, namely, extensive fibrosis, cirrhosis, and as further complication hepatocellular carcinoma (1, 2). 160 million people worldwide (2.35% from the general population) suffer from chronic hepatitis C infection. The social burden of HCV infection in 2007 exceeded that of HIV infection (3).

Majority of HCV infected patients is diagnosed at advance stages and may be present with the symptoms when they have already progressed to cirrhosis, increasing the risk for further development of hepatocellular carcinoma or need for liver transplantation (4). Evidences show that 10–20% of the patients with chronic HCV infection develop cirrhosis in the next 10–20 years, which leads to disability and low survival rates (5). Patients with HCV infection who already developed cirrhosis are at increased risk of hepatocellular carcinoma with annual risk of 3.7% (6, 7).

To present, there are no official data for the prevalence and mortality of HCV infection in Bulgaria and no clear data on the stage of the disease at the time of diagnosis. According to Bulgarian consensus on the treatment of chronic hepatitis C, the prevalence of HCV in the general Bulgarian population is 1.5%, but the majority of the patients are still not diagnosed (4). The advanced stage of diagnosing might result in high cost, especially due to hospital admissions (1).

Studies have shown that the cost of chronic HCV infection increases with the progression of disease severity. The delay in the treatment might result in disease progression, higher economic burden, and increased direct medical and societal costs (8). Major cost drivers are also hospital treatment of cirrhosis-related complications such as portal hypertension, ascites, spontaneous bacterial peritonitis, hepatic-renal syndrome, and encephalopathy (9–11). Therefore, studying the hospital cost of HCV-related cirrhosis is important due to the complex nature of the disease performance and significant comorbidity.

Lots of studies on the cost of HCV drug therapy have been published through the years evaluating the cost-effectiveness of the interferon-based dual and triple (boceprevir- or telaprevir-based) in different health-care settings (12–14). With the marketing authorization of the new direct-acting agents (DAA)-based interferon-free therapies, pharmacoeconomic analyses were also published studying their cost-effectiveness and budget impact compared to the current standard of care in different patients’ populations based on genotype and presence of HCV-related cirrhosis (15–19).

Analyses of the cost of HCV therapy at national level are scarce. In 2009, study on the cost-effectiveness of pegilated interferon alfa 2b was published, and (20) in 2012, a budget impact analysis on the HCV therapy after boceprevir addition to standard therapeutic regime in Bulgaria was performed (21). More analyses are done on the cost of chronic HBV infection (22, 23) and vaccination against HAV infection (24). There is no economic analysis on the cost of hospital treatment of patients with chronic HCV infection and cirrhosis and that motivated us to perform the current study.

The objective of this study is to perform analysis of the cost of therapy of patients with chronic HCV-related cirrhosis hospitalized in the University Hospital “Queen Joanna-ISUL” for 3-year period (2012–2014).

The point of view was that of the payers and patients.

We focus on the questions “What is the cost of hospitalized patients’ with HCV-related cirrhosis and how it is distributed among the disease severity and payers?”

## Materials and Methods

### Design of the Study and Patient Sample

Prospective, observational, 3-year lasting (2012–2014), non-interventional study of the real life therapeutic practice was performed. Consecutively, 301 patients were recruited with chronic hepatitis C infection-related cirrhosis, admitted at the Department of Gastroenterology in the University Hospital “Queen Joanna” and followed during the period of observation. These patients represent nearly 30% of all cirrhosis cases treated in the Department. The clinic is also a national center for treatment of liver tumor and leading national unit for endoscopy and treatment of chronic inflammatory liver diseases and admits patients from all the country. The Department of Gastroenterology leads a National Health Insurance Fund-funding programme for treatment of chronic hepatitis C and B infections with expensive medicines. Annually, the Gastroenterology Department is treating 1,000–1,200 patients with cirrhosis regardless the underlying condition.

Out of the 301 patients, 4 were excluded due to lack of information and 297 were analyzed.

Information about the clinical (Child-Pugh stage, ascites, portal hypertension, esophageal varices, encephalopathy, etc.), demographic (age and gender), and data on the utilized health-care resources was collected from patients files.

All patients fulfilled informed consent. Ethical Committee of the hospital approved the study.

### Patients’ Characteristics Analysis

The observed 297 patients were divided into subgroups depending on the severity of the cirrhosis according to Child-Pugh classification. During the 3 years, observational period patients did not change the critical clinical values for Child-Pugh status, and therefore the group was considered as homogenous. There were no patients for which physicians considered necessary to move from one to another Child-Pugh stage.

### Health-care Resources Utilization Analysis

Utilized health-care resources during the hospitalization period (cirrhosis drug therapy, therapy of cirrhosis-related complications, hospitalization resources, highly specialized procedures, and consumables) was recorded from the hospital patients’ records.

They were divided into three subgroups depending on the financing source [National Health Insurance Fund (NHIF), hospital, and patients’ out-of-pocket] and further subdivided according to Child-Pugh severity classification (Child-Pugh A, Child-Pugh B, and Child-Pugh C).

The subdivision based on the financing source was performed due to the specifics of the health-care financing system in Bulgaria. It is based on obligatory health insurance principles with patients’ co-payment for medicines and some of the ambulatory and hospital services. Medicines are reimbursed in Bulgaria when they are included in the positive drug list (PDL). PDL is divided into three annexes depending on the paying institution—Annex I (medicines paid by the NHIF for ambulatory therapy of chronic diseases), Annex II (medicines paid by the hospitals’ budget), and Annex III (medicines paid by the Ministry of Health for the treatment of HIV/AIDS, tuberculosis, malaria, infectious diseases, and vaccines). The medicines included in the Annex 1 of PDL are reimbursed according to ICD code of the diseases. The payment of hospitals is retrospective and covers preliminary negotiated procedures for the hospital stay of health insured patients.

### Cost Analysis

Micro-costing approach was applied to evaluate the direct medical costs for each group of patients depending on the Child-Pugh severity and from the perspective of the way of financing for the observed 3-year period.

Cost of resources was calculated by multiplying the unit cost of the resource by the number of patients utilizing it and the number of utilized units.

Total cost for each Child-Pugh subgroup is calculated as the sum of the cost paid by the hospital, the patient, and the NHIF for each Child-Pugh subgroup, using the following formulas:

$$\begin{array}{c} C_{\text{patients}}=\text{No. of patients utilizing the health}-\text{care resource}\times \text{Unit cost of the health}-\text{care resource}\times \text{No resources} \end{array}$$

$$\begin{array}{c} C_{\text{hospital}}=\text{No. of patients utilizing the health}-\text{care resource}\times \text{Unit cost of the health}-\text{care resource}\times \text{No resources} \end{array}$$

$$\begin{array}{c} C_{\text{NHIF}}=\text{No. of hospitalizations}\times \text{Unit cost of the hospitalization}\times \text{No resources} \end{array}$$

$$\text{Total costs}=C_{\text{patients}}+C_{\text{hospital}}+C_{\text{NHIF}}$$

Total cost for the observed 3-year period for all patients admitted to the hospital is derived as follows:

$$\begin{array}{c} \text{Total Costs}=\text{Costs for Child-Pugh A}+\text{Costs for Child-Pugh B}+\text{Costs for Child-Pugh C} \end{array}$$

Total costs is also presented as total cost/per patient for the observed period due to the differences in the number of patients assigned to each group according to Child-Pugh severity.

$$\begin{array}{c} \text{Total cost/per patient}=\text{Total cost/No. of patients}\;\text{in the particular Child-Pugh stage} \end{array}$$

The prices of the medicines were taken from the official register of the National council on pricing and reimbursement of medicinal products (25). The costs of the hospital stay and procedures were taken from the National Health Insurance Fund tariff (26). All costs were calculated for 3-year period in the national currency—Bulgarian leva (BGN). The exchange rate is fixed at the €1 = 1.95 BGN.

Cost of antiviral treatment was not included as the observed cohort of patients was contraindicated for the treatment with double therapy (pegilated interferon alfa 2b + ribavirin) and triple therapy (pegilated interferon alfa 2b + ribavirin + boceprevir or telaprevir) that were the accepted standard therapy during the period of observation. The new DAAs started to be available in the pharmaceutical market in Bulgaria later on since 2016.

### Statistical Analysis

Descriptive analysis was performed, correlation between the number of hospitalizations and the cirrhosis severity was explored through chi-squared test, Kruskal–Wallis, and Friedman non-parametric tests, and linear correlation and regression analysis were applied to examine the correlation between the cost per patient from the different paying perspective and the severity of cirrhosis.

## Results

### Patients’ Characteristics

Among the observed cohort of 297 patients, the majority were men (76%) and 24% were women in average age of 62 years (SD 10.38) (Table 1).

**Table 1:** Patients’ characteristics.

Indicator	Number	Average age (SD)
Total number of patients	297	62 (10.38)
Male (76%)	225	60 (10.35)
Female (24%)	72	62 (10.37)

Mortality rate during the observed 3 years was high—6.90%, most probably due to the asymptomatic progression of the chronic HCV infection. Patients in stage Child-Pugh A prevailed (Table 2).

**Table 2:** Distribution of patients according to the severity of the disease.

Indicator	Number of patients			Average age		Mortality rate		
	Men	Women	Total (%)	Men	Women	Men [number (%)]	Women [number (%)]	Number (%)
Severity of disease (Child-Pugh) at base line-A	120	48	168 (56)	63	62	6 (5.00)	3 (6.25)	9 (5.35)
Severity of disease (Child-Pugh) at base line-B	91	18	109 (37)	62	63	8 (8.79)	0 (0)	8 (7.34)
Severity of disease (Child-Pugh) at base line-C	14	6	20 (7)	54	61	3 (21.40)	1 (16.6)	4 (20.00)
Total	225	72	297	60	62	17 (7.55)	4 (5.56)	21 (6.90)

Gastrointestinal complications such as esophageal varices, ascites, and spontaneous bacterial peritonitis have been registered in most patients. All patients experienced more than one complication for the observed period. All complications have been considered as life threatening. The concomitant pathology mainly heart diseases and metabolite syndrome are presented due to the general impaired condition of the patients (Table 3).

**Table 3:** Cirrhosis-related complications and concomitant diseases in the observed cohort of patients.

	No. of patients	%
**Frequency and type of complication, diagnosed at baseline**		
Ascites	114	38
Spontaneous bacterial peritonitis	28	9
Other infection (urological, pneumonia, and soft tissue infections)	90	30
Encephalopathy	56	18.9
Esophageal varices 3–4 grade (incl. hemorrhage)	123	41.4
Esophageal varices 1–2 grade	174	58.6
**Concomitant pathology**		
Heart diseases	106	35.7
Metabolite syndrome/diabetes type 1 and type 2	108	36.4
Recurrent serious complications	62	21

On total 826 hospitalizations were reported for the observed cohort of 297 patients for the 3-year period with 17 days hospital stay per patient on average. The average number of hospitalizations per patient was 3 and followed tendency of change with the advancement of the Child-Pugh stage of cirrhosis, most probably due to the different number of patients in each subgroup (Table 4). Readmissions were not observed because no one of the patients was hospitalized within 1 month after the discharge.

**Table 4:** Number and type of hospitalizations depending on the health service.

Indicator	Total *N* units	Total *N* units Child-Pugh A	Total *N* units Child-Pugh B	Total *N* units Child-Pugh C
Total number of hospitalizations for 3 year	826	535	263	28
Average number of hospitalizations per patient	3.18 (SD 3.63)	2.41 (SD 2.36)	2.81 (SD 2.96)	1.4 (SD 0.68)

### Health-care Resources Utilization

More than half of the hospitalizations were due to decompensation of the cirrhosis. Other health-care resources that were utilized were procedures for complications from the peritoneum, pancreatic, and hepatic–biliary system and for ligation of varices.

Antibiotics’ prevail probably due to abdominal infections. Other prescribed medicines cover the cardiovascular complications (Table 5). National Health Insurance Fund covers the hospitalizations including procedures, physicians’ visits, and hospital charges. Antibiotics therapy with ciprofloxacin and ceftriaxone is mostly covered by hospital budget, and the rest of the medicines is subsidized by the patients because they are not included in the clinical recommendations for cirrhosis therapy. Norfloxacin is also paid out-of-pocket by the patients as it is not included into the positive drug list of Bulgaria and is not paid with public expenditures.

**Table 5:** Reasons for hospitalization and rate of utilization of health-care resources.

Indicator	No. (for 3 years)	Rate of utilization (for 3 years) (%)	Payer
**Hospitalizations by reason**	**No. of hospitalizations**		
Hospitalizations due to decompensated liver disease/cirrhosis	487	59	NHIF
Hospitalizations due to diseases of the hepatobiliary system, pancreas, and peritoneum	230	28	NHIF
Hospitalizations due to endoscopic variceal ligation	109	13	NHIF
**Medicines**	**No. of patients**		
Ceftriaxone	120	41	Hospital
Ciprofloxacin	111	38	Hospital
Terlipressin	12	4	Hospital
Esomeprazole	122	41	Hospital
Albumin	131	44	Patients
Spironolactone + furosemide	113	38	Patients
Propranolol/carvedilol	143	48	Patients
Norfloxacin	30	10	Patients
Bands for endoscopic treatment	45	15	Patients
l-Ornitine-l-aspartate	42	15	Patients
Contrast material	60	20	Patients

### Cost Analysis

#### Cost of Hospitalized Patients due to Disease Severity and Complications

**Figure 1:**
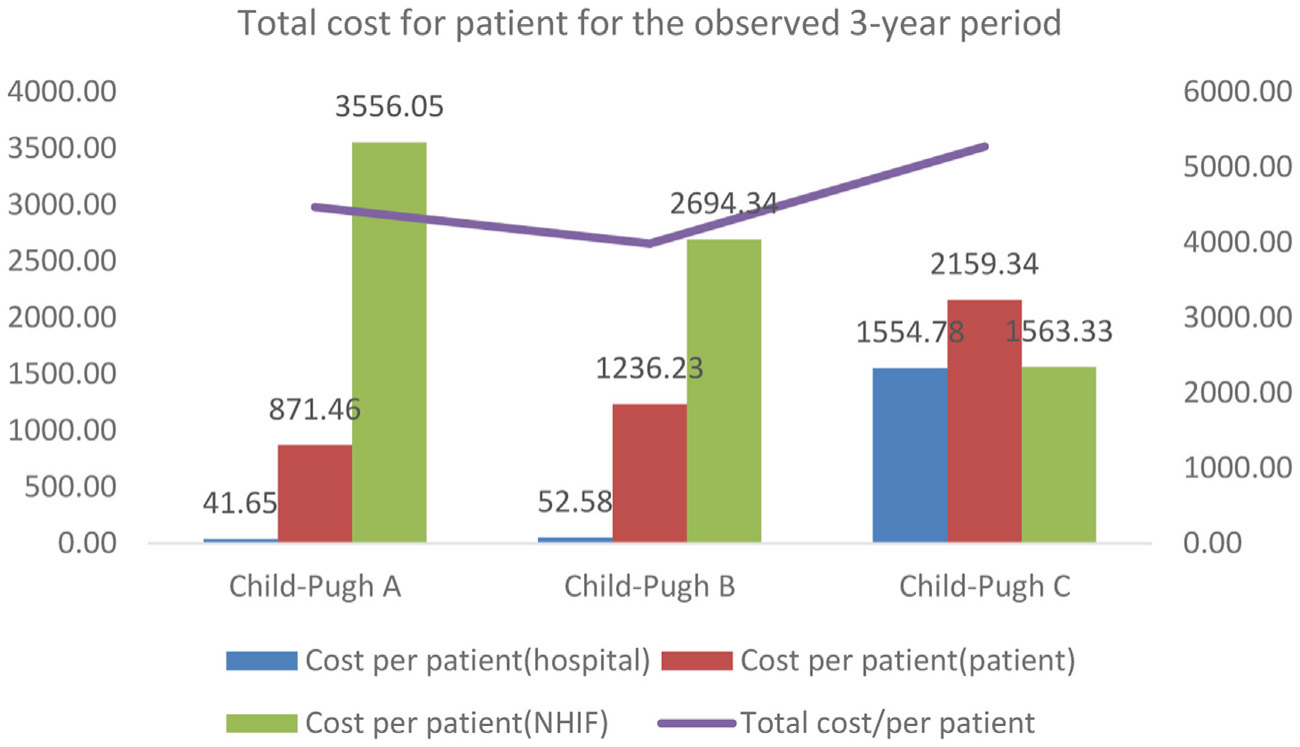
Distribution of costs for the observed 3-year period.

The total cost of hospitalized patients is presented from each payer’s perspective—hospital, patient, and NHIF. For the observed 3 years period, the total cost accounts for 1,290,533 BGN (€659,839). Per patient cost increased with advancement of the severity of disease as Child-Pugh category (Table 6; Figure 1).

**Table 6:** Total cost per patient for 3 years depending on the Child-Pugh severity.

	Cost per patient (from hospital perspective) (BGN)	Cost per patient (out-of-pocket) (from patient perspective) (BGN)	Cost per patient/NHIF (from NHIF perspective) (BGN)	Total cost/patient (BGN)
Child-Pugh A	41.65	871.46	3,556.05	4,469.17
Child-Pugh B	52.58	1,236.23	2,694.34	3,983.16
Child-Pugh C	1,554.78	2,159.34	1,563.33	5,277.45

The share of the cost per patient covered by the NHIF showed regressive tendency with the advancement of the disease, probably due to the small number of patients and related number of hospitalizations in the different subgroups (Figure 1).

From the perspective of the patients, the total cost/patient is almost doubled for the patients with Child-Pugh C cirrhosis compared to patients with Child-Pugh A. The cost/patient from the hospital perspective is significantly higher for patients in the severe stages of cirrhosis compared to the mild and moderate stages.

### Statistical Analysis

Statistically significant moderate correlation exists between the number of hospitalizations and the Child-Pugh stage (*P* = 0.0237).

Positively are correlated also the time period for follow-up (measured as time in months for monitoring after hospital discharge) and the Child-Pugh stage (*P* = 0.0039). Patients with Child-Pugh stage B are followed for a longer period than patients in stage A thus also consuming higher cost. Stage Child-Pugh C is not included due to small number of patients.

The *post hoc* analysis of the non-parametric Kruskal–Wallis test showed that there is a statistically significant difference in the total cost per patient from the different payers’ perspective (hospital, patient, and NHIF) (Table 7).

**Table 7:** Kruskal–Wallis test results.

Factor	*n*	Average rank	Different (*P* < 0.05) from factor nr
**Total cost/patient (hospital perspective), *P* = 0.000001**			
(1) Child-Pugh A	168	132.55	(2) (3)
(2) Child-Pugh B	109	158.93	(1) (3)
(3) Child-Pugh C	20	233.10	(1) (2)
**Total cost/patient (NHIF perspective), *P* = 0.006343**			
(1) Child-Pugh A	168	160.09	(3)
(2) Child-Pugh B	109	139.60	
(3) Child-Pugh C	20	107.05	(1)
**Total cost/patient (patient out-of-pocket perspective), *P* = 0.000027**			
(1) Child-Pugh A	168	133.77	(2) (3)
(2) Child-Pugh B	109	159.55	(1) (3)
(3) Child-Pugh C	20	219.45	(1) (2)

The Friedman test showed that there is statistically significant correlation between the total cost per patient from the different payers’ perspective in Child-Pugh A and Child-Pugh B groups but not for Child-Pugh C, most probably due to the very low number of patients in this group—only 20 (Table 8).

**Table 8:** Freedman test results.

Variable	Mean rank	Different (*P* < 0.05) from variable nr
**Child-Pugh A, *P* < 0.00001**		
(1) Total_cost_hospital	1.3214	(2) (3)
(2) Total_cost_patient	1.8512	(1) (3)
(3) Total_cost_NHIF	2.8274	(1) (2)
**Child-Pugh B, *P* < 0.00001**		
(1) Total_cost_hospital	1.2339	(2) (3)
(2) Total_cost_patient	2.0780	(1) (3)
(3) Total_cost_NHIF	2.6881	(1) (2)
Child-Pugh C, *P* = 0.8814		

**Figure 2:**
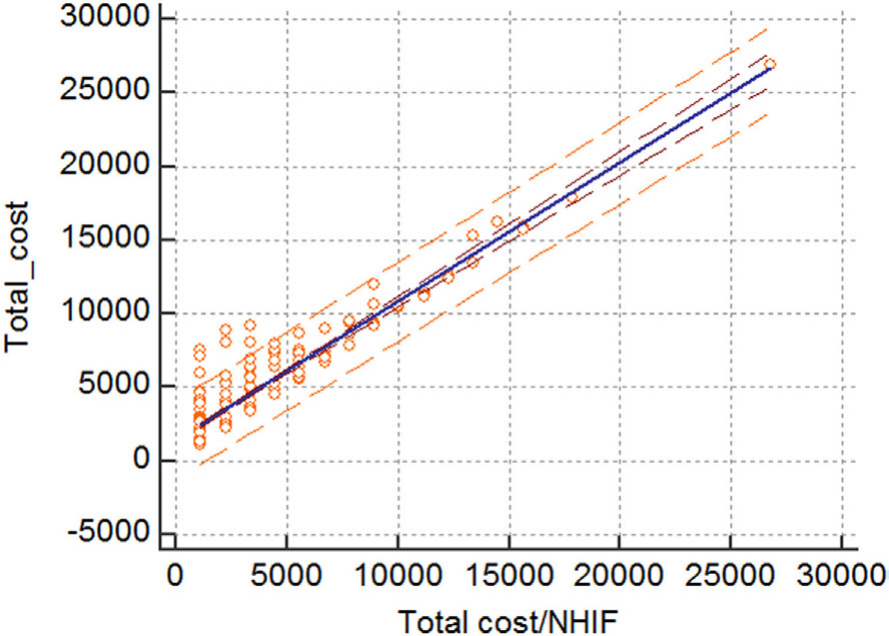
Linear regression correlation.

The linear correlation and regression analysis showed that there is statistically significant linear correlation and regression between the average total cost from NHIF perspective and the average total cost of treating patients. 83.9% of the variance of the average total cost is due to the changes in the average cost paid by the NHIF. This could mean that the NHIF costs are major cost drivers in the hospital treatment of patients with HCV-related cirrhosis (Figure 2).

## Discussion

Hospital treatment of HCV-related cirrhosis induces high utilization of health-care resources and high costs that significantly contributes to the economic burden of the chronic HCV infection. In comparison, other national studies on chronic diseases also confirm that the cost of the hospitalizations due to related complications is one of the major cost drivers. Economic analysis on the treatment of chronic HBV infection shows that the average hospital cost per chronic case related to cirrhosis is 712 BGN (24). Cost analyses on the treatment of diabetes in Bulgaria show that the ambulatory cost per patient with diabetes on insulin analog therapy for a 6-month period is 652 BGN, which account for 46% of the total cost on average while the hospitalizations costs for complications due to diabetes are 23% of the total costs (27). Diabetes type 2 patients were associated with higher average cost per hospitalization due to complications (1,273 BGN) compared to patients with diabetes type 1 (933.26 BGN) (28). Currently published cost analysis on the treatment of COPD patients in Bulgaria showed that the average annual cost per patient of health-care utilization is 1,132 BGN and that of pharmacotherapy—1,355 BGN (29). An increasing trend in the cost of hospitalizations due to exacerbations was also observed from 2009 to 2014 (30).

Nevertheless, studies on the cost of treatment of HCV-related complications are scarce, all showed that HCV-related cirrhosis significantly increases the hospitalizations, the cost of medicines therapy, and the mortality rate (15–21, 31, 32) One study conducted in Spain examined through modeling techniques, the HCV-related complications and their costs (33).

A cost study performed in Belgium showed that in mild stage of the disease, the costs were €18,993 and increased 1.6 times with decompensated cirrhosis (€29,759), 1.9 times with hepatocellular carcinoma and 3.4 times with liver transplant (€65,120). In early disease stages, the main cost driver was the medicines cost, while with progression of the disease the cost of hospitalizations contributed from 8% of the total costs in decompensated cirrhosis to 79% in post liver transplantation. In contrast, our study recovers sixfold increases in total cost from Child-Pugh A to C stage probably because of the late diagnosis.

Two US-based studies evaluating the economic burden of the chronic HCV infection examine the impact of severity on the health-care costs. The cost analysis performed by Gordon et al. showed that HCV-related monthly costs per patient for compensated cirrhosis and end stage liver disease were 32 and 247%, respectively, higher than the costs in patients without cirrhosis (8). The other study, performed by McAdam-Marx and colleagues, showed that the incremental per-patient per-year costs increase significantly to $5,870 for patients with compensated cirrhosis, $27,845 for decompensated cirrhosis, $43,671 for hepatocellular carcinoma, and $93,609 for liver transplant compared to patients without the disease (*P* < 0.001) (34). Transferring the results of these studies to our patients means that with the progression of cirrhosis, we might expect further increase in the total cost.

Canadian study estimates the HCV-related disease and costs. Combined retrospective–prospective study projects the prevalence and the related costs of HCV chronic infection and its complications. The results show that the total health-care costs, excluding the treatment, associated with HCV, are expected to increase by 60% from 2013 until 2032. The majority of the expenditures are projected to be attributable to cirrhosis and its complications (81% in 2032 vs 56% in 2013) (35).

These are studies addressing the cost of cirrhosis in terms to its compensation status. Other studies examine the changes in the cost with its Child-Pugh progression. Study performed in Mexico examines the annual cost of treating patients with cirrhosis according to the Child-Pugh stage. Results show that cost of cirrhosis management is considerable and increases with the severity of the disease (36). These finding are in line with the results from the present study.

For our study, we chose to evaluate the changes in the cost according to the Child-Pugh stage as this score could be also used as a predictor for economic outcomes (37).

Nevertheless, antiviral treatment of chronic HCV infection is a major cost driver in the total cost of treatment of these patients in our study, these costs are excluded as the patients were contraindicated for antiviral treatment with the current therapeutic standard of double and triple therapy at the time of observation.

The results of our study could be used as a base case scenario before the entrance of the novel therapy in the Bulgarian pharmaceutical market. Further studies need to be done to calculate the cost of cirrhosis therapy after the introduction of the new antiviral DAAs for treatment of chronic HCV infection in Bulgaria.

To the best of our knowledge, this is the first study focusing on the cost of treatment of HCV-related cirrhosis in Bulgaria. This is also the first micro-costing study that evaluates a patient sample for a period of 3 years. The study shows that the progression of the chronic HCV infection and the development of cirrhosis as well as cirrhosis-related complications induce higher costs of treatment and follows the trend presented by other published costs studies on the economic burden of HCV-related complications and other chronic diseases with social burden.

The decompensation of the cirrhosis led to increased mortality rate in the observed cohort of patients due to direct impairment of the liver function. These findings are in line with other published results showing that the worsening of the disease leads to increased morbidity and mortality, especially in patients suffering from varices (38).

The cost analysis also shows that expenditures are made not only by the National Health Insurance Fund but also from the hospital and from the patients as well. Patients even bear the prevailing part of the financial burden, especially for medicines therapy. Advancement of the disease let to increase in the cost per patient.

In this study, we triple tested the statistical significance of the relation between the cost and severity of cirrhosis (chi-square, Freedman, and Kruskal–Wallis tests) due to the low number of patients in Child-Pugh C stage. All tests confirm that the severity impacts the number and cost of hospitalizations significantly. In addition, the correlation model finds the NHIF cost as a major cost driver.

It must be noted that our study has a few limitations, because it focuses mainly on the most visible part of the public and patients’ expenditures. The costs for caregivers and outpatient treatment, concomitant diseases treatment, etc., are not included in the cost analysis, as well as the cost of ambulatory pharmacotherapy of cirrhosis and other comorbidities, which is reimbursed by the National Health Insurance Fund. Also it should be noted that this study is based on the current therapeutic practice. HCV-related cirrhosis is treated according to EASL guidelines and the national management standards. In our patient cohort, we did not include cirrhosis related to other types of hepatitis or diseases, and we cannot comment any differences in the treatment and management.

## Conclusion

HCV-related cirrhosis is resource demanding and induces high direct medical costs as it is related with lots of hospitalizations and leads to complications acquiring additional treatment. Cost is increasing with the progression of the disease, and patients bear significant amount of the total financial burden.

## Ethics Statement

The observational protocol was reviewed and approved by the Ethical Committee of the University Hospital “Queen Joanna-ISUL.”

## Author Contributions

GP from the Medical University in Sofia contributed to the cost data revising, statistical analysis review, and writing. She is a leading pharmacoeconomic expert in Central and Eastern Europe. JG is a key opinion leader in the field of hepatology in Bulgaria. His contribution is related to the revision of the collected patients’ demographic and clinical data, giving conclusions and revising the manuscript. KP contributed to the collection and creation of the patient database and writing the manuscript. KM conducted the statistical analysis. MD’s contribution to this work is in cost data analyzing, statistical analysis review, and writing.

## Conflict of Interest Statement

The authors declare that the research was conducted in the absence of any commercial or financial relationships that could be construed as a potential conflict of interest. The reviewer, AAAS, and the handling Editor declared their shared affiliation, and the handling Editor states that the process nevertheless met the standards of a fair and objective review.
